# Wireless Devices for Optical Brain Stimulation: A Review of Current Developments for Optogenetic Applications in Freely Moving Mice

**DOI:** 10.1007/s12195-024-00832-z

**Published:** 2024-11-22

**Authors:** Patrícia Silva, Luis Jacinto

**Affiliations:** Department of Biomedicine – Experimental Biology Unit, Faculty of Medicine of the University of Porto (FMUP), 4200-319 Porto, Portugal

**Keywords:** Optogenetics, Wireless devices, Optoelectronics, Rodent behavior, Neural modulation

## Abstract

**Purpose:**

Optogenetics is an invaluable tool to study brain circuits, but typical systems rely on tethered approaches to deliver light to the brain that hinder natural behavior. With the increasing prevalence of complex behavioral phenotyping in neuroscience experiments, wireless devices for optical stimulation offer great promise to overcome these limitations.

**Methods:**

In this work we critically review recent systems engineering and device design approaches to deliver light to the brain with wireless operation for optogenetic experiments.

**Results:**

We describe strategies used for wireless control and communication, wireless power transfer, and light delivery to the brain with a focus on device integration for in vivo operation in freely behaving mice.

**Conclusion:**

Recent advances in optoelectronic systems, material science, and microtechnology have enabled the design and realization of miniaturized wirelessly-controlled optical stimulators for true untethered experiments in rodent models.

## Introduction

Optogenetics is a research tool that combines genetic manipulation of cells with photonics for control of cell activity with light [1]. Its application in neuroscience relies on the expression of light-gated ion channels and pumps, that are activated by specific light wavelengths, under the control of cellular promoters found on brain cells [2]. This cellular specificity allows that different types of cells, brain areas, circuits, and neuronal connections can be excited or inhibited by electrochemical currents driven by light stimulation [2–5]. Because cells can be genetically modified to express different light-gated proteins, cellular control can occur at multiple wavelengths in the same cells but also at different brain areas. When compared with other forms of brain stimulation used in basic and preclinical neuroscience experiments, such as electrical or electromagnetic stimulation, optogenetics has the advantage of allowing high spatiotemporal precision with reversible control on top of the cellular specificity. On the other hand, its disadvantages include requiring viral transduction or genetic editing for the expression of the light-gated channels in neuronal cells, and invasive light delivery systems or devices [2, 6]. Nevertheless, optogenetics has allowed researchers to dissect the functional role of brain circuits more efficiently over the last decade and to highlight potential therapeutic targets for brain disorders [5, 7, 8].

Conventional neuroscience experiments using optogenetics typically rely on optical fiber implants and steady-state laser sources to deliver the light to the brain for optical stimulation [9–11]. However, these systems can be costly and require tethered approaches to connect the light sources to the optical implant on the animal which can hinder natural behavior and assessment of behavioral outcomes. Therefore, in recent years, there has been increased interest in devices for optical stimulation in optogenetic studies that can be wirelessly controlled. Advances over the last decade in optoelectronic systems and their miniaturization, including novel materials and microfabrication strategies [12], as well as improved wireless communication and power transfer approaches [13–15], allowed the emergence and progressive refinement of wireless optical stimulators for biomedical applications, including optogenetics.

In this review we provide an extensive description and critical assessment of developments in wireless devices for optical brain stimulation, with a focus on device integration for optogenetic applications in freely-moving mice.

## Wireless Optical Stimulators

**Fig. 1:**
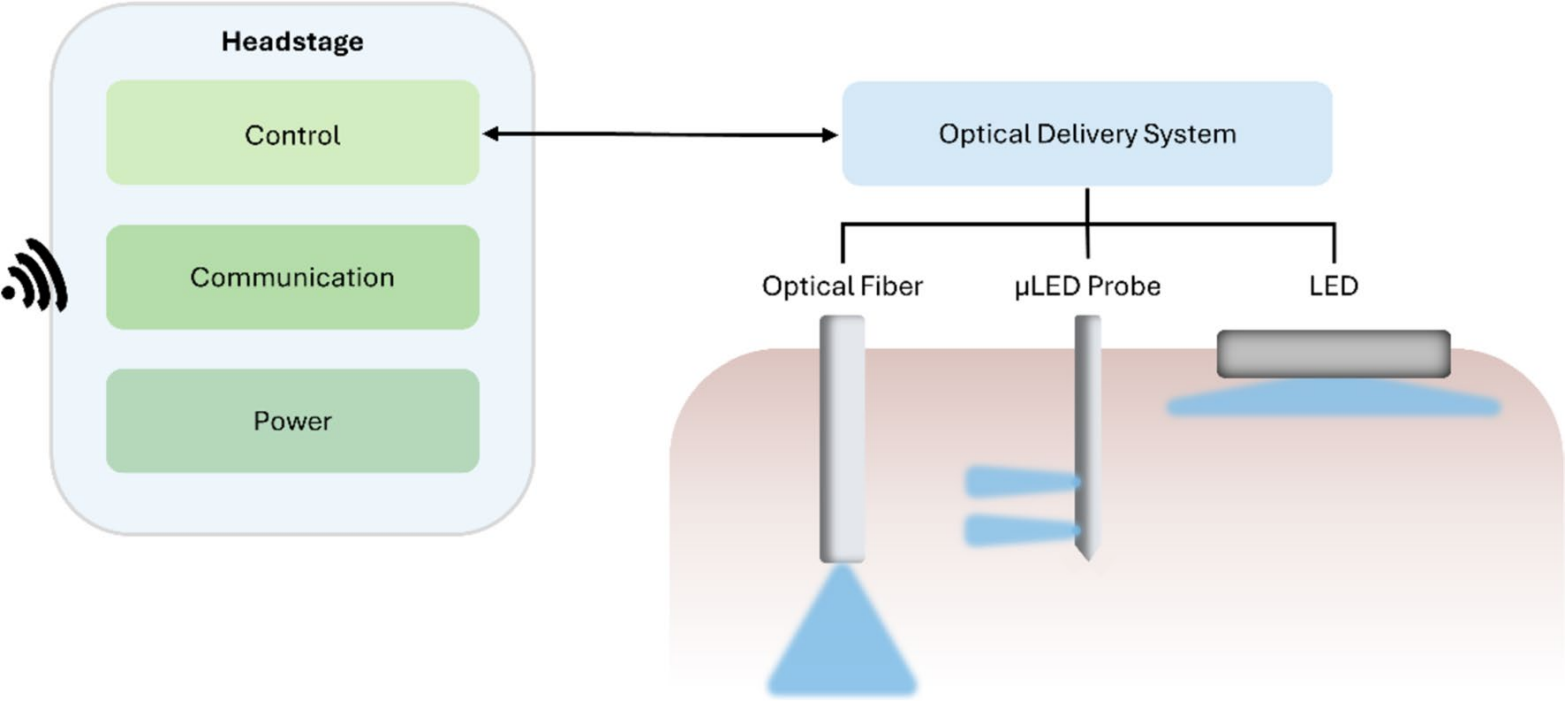
Overview of a general wireless optical stimulator for optogenetics experiments. Schematic of the principal components of the headstage with control, communication, and power modules (left). The headstage communicates wirelessly with an emitter, receiving control inputs. Typically, headstages also include a power module, in the form of an independent source (such as a battery) or a wireless receiver for wireless power transfer. The headstage is physically connected to a light source that can deliver light to the brain through different strategies (right). These include transcranial illumination through LEDs or intracerebral illumination through optical fibers or implantable micron-sized probes containing µLEDs

The devices here reviewed present different structural designs and modes of operation, but all require control electronics for wireless communication and/or stimulation triggering. In general, the devices described can be divided into two main parts: the headstage, which typically integrates control and communication electronics but can also include components for receiving wirelessly transmitted power; and the optical delivery system, including optical fibers or light-emitting diodes (LEDs), which can be implantable or not. Figure 1 summarizes the different optical delivery strategies used on the devices reviewed, as well as a schematic for the principal components of the headstage. This review divides optical stimulators for optogenetics experiments in mice according to the mode that light is delivered to the brain. The different sections describe transcranial, fiber-coupled, implantable micro-scale LEDs (µLEDs), and multifunctional optical stimulators. The characteristics of the wireless optical stimulators reviewed are summarized in Table 1.

**Table 1:** Wireless optical stimulators for freely moving mice optogenetics experiments

References	Optical delivery	Stimulation type	Headstage location	Communication	Range (m)	Power	Battery lifespan (min)	Operating voltage (V)	Irradiance (mW/mm^2^)	Stimulation wavelength (nm)	Weight (g)	Footprint	Customizable parameters	GUI
[20]	LEDs	Transcranial	External	IR	2.0	Battery		5.0			3.1	25 × 7.5 mm		
[21]				RF	1.0	RF					3.0 / 2.0	19 × 19 × 8 mm	X	
[22]				IR	15.0	Battery	64	5.0	6.1		2.4	14 × 14 × 11 mm		
[25]	Fiber-coupled	Intracerebral	External	RF	1.0			4.0	27.0		1.6	12 × 5 × 11 mm		
[26]				IR	1.8		55	3.7	4.2		2.8	15 × 20 × 10 mm	X	X
[27]				EM					2.8		1.5	< 1 cm^3^		
[30]	µLED	Intracerebral	External	RF	4.0	Battery	120			465	2.9	14 × 17 mm	X	X
[31]				RF	0.3	RF			20.0		0.02–0.05	10 mm^3^	X	
[32]			Subdermal	RF	0.3				50.0	470	0.03	10 mm^3^	X	
[35]			External	RF	50.0	Battery	240		82.0	471	1.9	10 × 18 mm	X	
[36]				RF			120		50.0/200.0	630/480	1.9	10 × 18 mm	X	
[38]				RF		RF			2.3		0.7	3.5 × 2.4 × 8.5 mm		
												5 × 12 mm		
[40]				RF		Solar			12.0		0.03	4.3 × 8 × 0.7 mm		
[42]			Subdermal	RF		RF		2.5–3.5		475	15/60/75 × 10^-3^		X	
[43]		Transcranial		RF		RF			226.6	628	0.076	11.73 x 7.95 x 0.39 mm	X	
[44]		Intracerebral		RF		RF			30.0	460/535/595 / 630		10 × 12/11 × 19 mm	X	X
[46]			External	BLE		RF	720	2.3–2.8	5.0		2.2/3.2	D: 14 mm	X	X
[47]							Solar-recharge	3.8	40.0	460			X	
[39]	µLED & temperature	Intracerebral	External	RF	1.0	RF			7.0	450	0.7 / 2.0			
[48]	µLED & pharmacological			IR		Battery					1.85	1575 mm^3^		
[49]			Subdermal	RF	0.1	RF			30.0		0.22	125 mm^3^		
[50]			External	BLE	100.0	Battery				470 / 589	2.0	1260 mm^3^	X	X
[51]				RF		RF			4.2–33.7		0.29	78.5 mm^2^ × 4 mm	X	X
[52]	µLED & dopamine					Battery				470	2.0	22 × 13 mm		

*BLE* bluetooth low energy, *D* diameter, *EM* electromagnetic, *GUI* Graphical User Interface, *IR* infrared radiation, *RF* radio-frequency, *V* voltage, *µLED* micro-scale light emitting diode

### Transcranial Optical Stimulators

The early systems for rodent in vivo brain optogenetics typically used tethered implantable (or cannula-guided) optical fibers to deliver light from a steady-state laser source to the brain [16–18], but there were also approaches using surface illumination of superficial cortical layers by directly applying the light from the laser through a patch cable or a tethered LED to the brain or through the skull [19].

One of the first reported wireless stimulators for cable-free in vivo optogenetics used a transcranial stimulation approach. Iwai et al. proposed in 2011 a simple battery-powered printed circuit board (PCB) with a through-hole blue dual in-line package (DIP) LED that could be anchored to the skull and deliver light through a thinned skull [20]. Wireless communication to trigger light pulses in the headstage was achieved with an infrared radiation (IR) transceiver with multiple IR-LEDs to enable IR transmission to the receiver at any angle up to 2 m range. The receiver was powered by two lithium polymer-based (LiPo) batteries, weighing 0.7 g each, and it was operational with 5 and 6 V. The maximum output power of the LED was of 7 mW, but stability decreased down to 5 mW with repetitive pulses. At 3.1 g, the device was above the expected weight limit to be used in mice, but it was effectively tested in two Thy (thymus cell antigen)-ChR2 (channelrhodopsin-2)-YFP (yellow fluorescent protein) mice during freely moving behavior. Although this device was an important early demonstration that a wirelessly-controlled device could provide effective optical stimulation to the rodent brain, the use of conventional though-hole and hand-soldered large components in the PCB contributed to its limiting bulkiness (25 × 7.5 mm) and weight.

In the same year as [20], Wentz et al. developed a wireless modular system for transcranial optogenetic stimulation but using an array of surface-mount device (SMD) LEDs and with wireless power transfer (WPT) capabilities [21]. The headstage included four distinct modules: power, communication, motherboard, and optical. The communication module was based on radiofrequency (RF) transmission in the 2.4–2.485 GHz band. The power module used a resonant RF approach and included a 16 mm long antenna, alternating current (AC)/direct current (DC) conversion by a rectifier, and a supercapacitor to store transmitted power. The WPT range limited the distance between the base and the animal’s head to below 1 meter. The motherboard module included a microcontroller for device operation and the LED power conditioning circuit. The optics module, the only to be permanently affixed to the skull, included an array of 16 bare die LEDs and a thermistor to monitor optical-induced temperature elevations in the tissue (that de-activated the LED whenever the temperature increase surpassed 1 ºC). All other modules were connected for behavioral experimental sessions only, and the communication module could be omitted in case of previously programmed stimulation parameters for autonomous operation. The total weight of the device was 3 g with the communications module, and 2 g without it, with an estimated footprint of 19 × 19 × 8 mm. A USB-connected base station was designed to communicate with the radio chip to deliver triggers on demand and to program the microcontroller before placing the headstage on the animal’s head. Although the optical power of the LED array was not reported, each LED was powered by 250 mW. The device was tested on untethered mice expressing ChR2 in the motor cortex freely exploring a 20-cm diameter acrylic arena positioned on top of the wireless power transmitter. Although successfully tested in mice, the device weight and bulkiness were still high, limiting experimental conditions. An additional limitation was that the effective distance was dependent on the short range of the WPT system and required small behavioral arenas to be placed on top of the WPT transmitter. Although not requiring programming skills, users had to interact with a terminal, introducing commands, to program stimulation parameters and trigger the stimulator remotely.

**Fig. 2:**
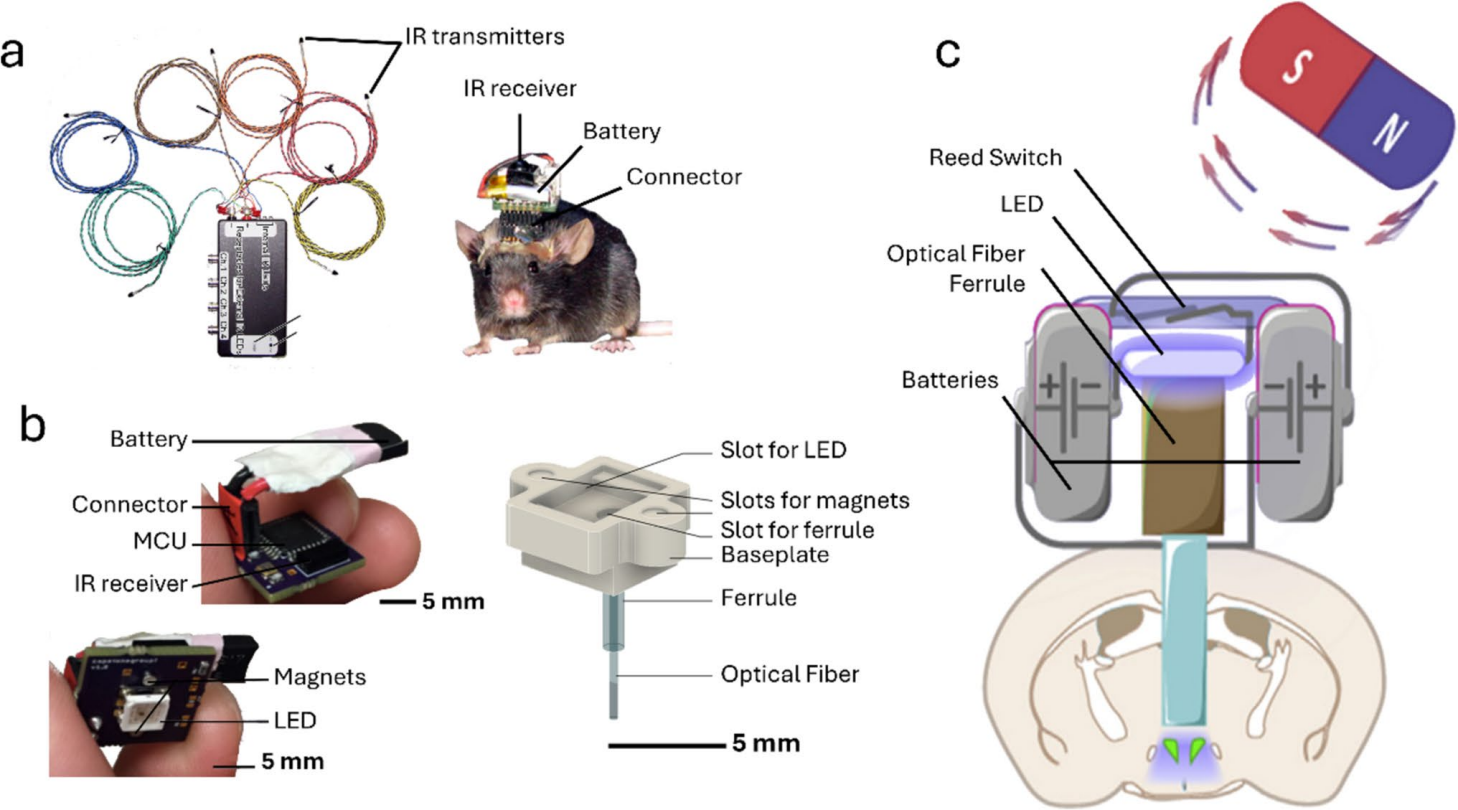
Wireless stimulators using LEDs for brain illumination. **a** Wireless device for transcranial optical stimulation that communicates with base station transmitter with multiple parallel transmitters though infrared radiation (IR) (left). The headstage contains an IR receiver and battery and is coupled to an LED permanently on the skull through pin connectors (right) (adapted from [22] (CC-BY)). **b** Fiber-coupled device for wireless stimulation with an headstage consisting of a battery, microcontroller (MCU), LED, and an IR receiver (left). The headstage couples to a baseplate permanently affixed to the mouse’s skull through magnets. The baseplate holds an optical fiber implanted in the mouse’s brain (reproduced from [26] (CC-BY)). **c** Wireless optical stimulator controlled with electromagnetics (EM). The headstage contains two batteries, an EM-activated reed switch, an LED, and a ferrule-coupled optical fiber. This device is permanently attached to the mouse’s skull (reproduced with permission from [27])

Another device using transcranial illumination by LEDs and wireless control via IR signals was proposed by Hashimoto et al. in 2014 [22] (Fig. 2a). The stimulator was a small PCB containing a microcontroller, an IR receiver, pin connectors for up to 3 LEDs, a 10-mAh LiPo battery (0.52 g), and a step-up DC-DC converter (to convert the battery 3.7 V to 5 V). A custom 38 kHz IR transmitter with 6 independent IR channels allowed the simultaneous control of 6 independent headstages, within a range of 15 m, where individual 8-bit binary codes were matched between emitter and headstage. The LEDs (1.6 × 0.8 × 0.68 mm) were directly fixed to the skull and connected by wires to pin connectors, also anchored to the skull with screws and dental cement. Optical irradiance from each LED was of 6.07 mW/mm^2^, with a maximum of 1.8 mW/mm^2^ reaching the surface of the brain through the skull. The wireless stimulator, weighing 2.4 g and measuring 14 × 14 × 11 mm, including the battery, was connected via pin sockets to the skull’s pin connectors for behavioral experiments. Battery lifetime was approximately 3.5 h in standby operation and 1 h when driving the LED stimulator continuously at 10 Hz with maximum output power. The device was tested in vivo in Thy1-ChR2-YFP transgenic mice performing motor exploratory behavior with brain surface stimulation of motor and sensory cortical areas (M1, mlV2, and pM1/S1). This device presented a significant improvement in operational range and a reduced weight compared to [20, 21], facilitating mice experiments. Additionally, it used IR for communication instead of RF, which is a simpler technology to recreate, requiring fewer electronic components. However, the device by Wentz et al. [21] offered customizable stimulation parameters, which allowed more flexibility in experimental design.

### Fiber-Coupled Optical Stimulators

Delivery of light to deeper brain structures with transcranial or surface LEDs is challenging without using very high power, as optical power decreases as a function of light source distance [23, 24]. This requires that wireless devices can provide enough power to drive the high-brightness LEDs, and increases in temperature at the brain surface can potentially lead to brain damage or altered brain activity. Thus, other initially developed wireless optical stimulator devices used fiber-coupled approaches. This strategy has the optical fiber permanently implanted inside the brain to deliver light to a specific brain area and coupled to the headstage through means of ferrules. This approach is closer to the majority of the tethered systems used for in vivo optogenetics experiments where the implanted optical fibers are coupled with ferrule connectors to an external laser source or LED via patch cables [9–11].

The first wireless device using an optic fiber-LED coupling approach was proposed by Lee et al. in 2015 [25]. The wireless stimulator had a modular design with three modules: power, communication, and optical. The optical module consisted of a SMD blue LED embedded on the top side of a PCB and an optical fiber ferrule mounted on the bottom side. Coupling between LED and fiber was done via a through-hole in the PCB which also secured the fiber in place. This module was the only permanently affixed to the animal’s skull. The communication module used RF, up to 1 m range, but just for external triggering to wake the microcontroller and deliver a pre-programmed stimulation protocol. Programming of the microcontroller was achieved by a 10-pin serial wire debug (SWD) interface. The power module consisted of two SMD solid-state rechargeable batteries. Maximum optical irradiance at the optical fiber tip (200 μm, 0.39 NA) was of 27 mW/mm^2^. Even though the stimulator headpiece was composed of multiple stacked PCBs, one per each module, it had a total weight below 1.6 g and a footprint of 12 × 5 × 11 mm. In vivo validation was performed in ChR2-expressing mice with stimulation of the ventral tegmental area (VTA) during a conditioned place preference test. Even though this was the first wireless stimulator for optogenetics to explicitly allow control of the optical output power, this had to be pre-programmed into the microcontroller and not via RF communication. Nevertheless, the use of an LED-optical fiber coupling approach allowed in vivo modulation of brain activity of a deep brain structure by a wirelessly-triggered optical stimulator for the first time.

Another fiber-coupled wireless stimulator, CerebraLux, was proposed by Dagnew et al. in 2017 [26] (Fig. 2b). This device was composed by two modules: electronic and optic, the latter fixed on the mouse's skull with only 0.3 g. The two modules connected to each other through magnets, which facilitated positioning and removal of the headstage while strongly securing it in place during behavioral experiments. The optics module consisted of a high-density polyethylene (HDPE) baseplate with a ferrule-connected optical fiber fixed across a through-hole (similar to the approach in [25]). The electronic module included a SMD IR communication component, a microcontroller and a removable LiPo battery. IR communication allowed setting stimulation protocol parameters and remotely trigger the stimulation, with a maximum range of 1.8 m. This device also allowed setting the output power through pulse width modulation (PWM). The maximum optical irradiance at the tip of the optical fiber (500 μm, 0.63 NA) was 4.2 mW/mm^2^. The total weight of the device was 2.8 g, with the battery contributing with 1 g. The footprint dimensions of the PCB holding the IR module, microcontroller, and battery was approximately 15 × 20 × 10 mm. The battery lifetime was measured to be 55 min when performing continuous 10 Hz stimulation at maximum output power. A Python-based graphical user interface (GUI) was also developed to permit user-friendly setting of stimulation parameters and the IR transmitter was controlled by an Arduino. The device was tested in vivo with Ai27xD1-cre mice in a motor exploration task. Although this device allowed remote programming and triggering of stimulation protocols, the 1.8 m range was short, with the authors reporting that fluorescent lighting, frequently used in animal facilities, interfered with the IR communication.

A recent approach also using an optical fiber coupled to a LED was presented by Anpilov et al. in 2020 [27] (Fig. 2c). This device was only composed by two batteries connected in series, a blue LED, and a reed switch that was activated whenever a magnetic field was applied. Thus, it used none of the wireless communication strategies described above, relying only on the proximity of a magnetic field to turn the LED on. This allowed placing an electromagnet in specific behavioral arena positions for wireless control of optogenetic stimulation in spatially-defined arena zones. The final device weight was only 1.5 g, including the dental cement for skull fixation, due to the few electrical components used, and had a volume below 1 cm^3^. The maximum reported optical power at the fiber tip (400 µm) was 2.8 mW/mm^2^. This device was tested in vivo in Ires-Cre-*Oxt:Ires-Flp-Avp* mice, where continuous brain stimulation occurred only when the mice explored a feeder apparatus in a specific zone of a complex semi-natural behavioral arena. Although this device presented an ingenious approach to wirelessly-control optical stimulation in large size arenas, including for semi-natural and natural analysis of mice behavior, it did not allow wireless triggering on demand or modulation of the stimulation parameters.

### Implantable µLED Optical Stimulators

Fiber-coupled devices suffer from light loss at the LED-fiber interface due to free-space refraction and the uncollimated nature of LEDs light emission. This means that most of the output power is lost at the interface, reducing the efficiency of the devices, the battery lifetime, and the amount of light that effectively reaches the brain tissue. With the progressive miniaturization of LEDs came the idea of using implantable μLEDs that could be directly controlled by headstages’ generated currents [28, 29]. For chronic in vivo applications, µLEDs can be mounted on implant size shanks/needles or directly microfabricated on silicon or polymeric substrate neural probes [30–32]. Although µLEDs can increase the wall-plug efficiency of the devices, i.e. the amount of input electrical power that is converted into optical power, when compared with coupled LED-fiber approaches, a part of the power is lost due to heat dissipation in the electric tracks, contact pads and the µLED. Because temperature increases above 1º C can lead to thermally induced brain activity [33], it is especially important to assess heat dissipation as a function of output in these devices.

**Fig. 3:**
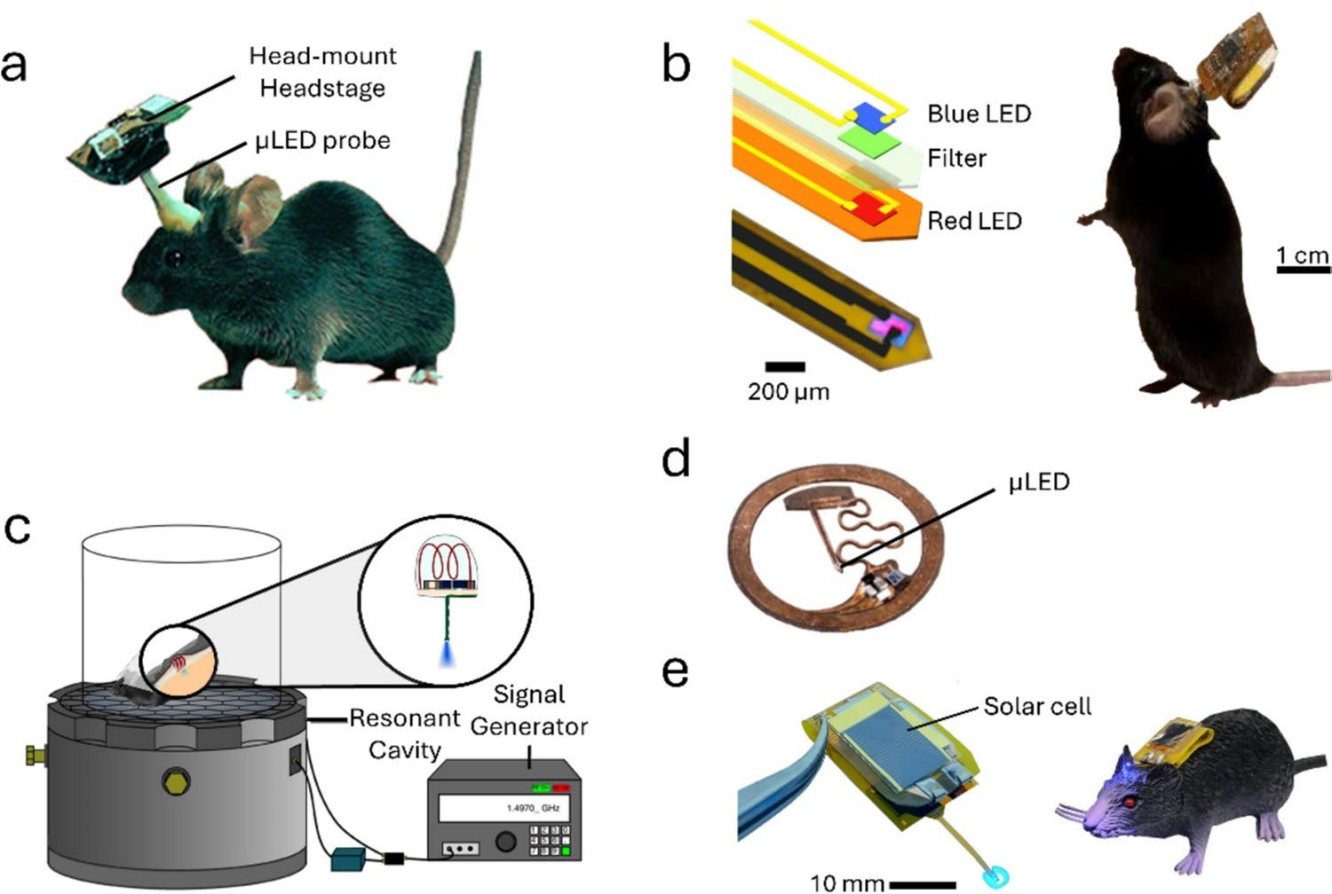
Wireless stimulators using neural probes with µLEDs to deliver light to the brain. **a** wireless optical stimulator with a head-mounted headstage consisting of a microcontroller, built-in memory, RF receiver, and a rechargeable battery. The headstage is connected to an implantable reduced-thickness PCB with a blue µLED at the tip (from [30] (CC-BY)). **b** Device with RF communication and powered by a battery, connected to a flexible polymeric neural probe with two co-localized µLEDs with different colors for dual opsin stimulation (from [36] (CC-BY)). **c** System for wireless power transfer and triggering of a wireless optical stimulator. The headstage consists only of a copper coil and a rectifier circuit permanently connected to an implanted blue µLED. The system transfers power to the headstage by induction though a resonant cavity with a lattice of hexagons (reproduced with permission from [31]). **d** RF-powered and controlled wireless optical headstage fabricated in a flexible substrate that can be implanted subdermally. The headstage is permanently connected to a flexible polymeric neural probe with a µLED through a serpentine cable (reproduced with permission from [42]). **e** Foldable wireless headstage for optical stimulation with a solar cell for battery-charging. The headstage is fabricated on a flexible PCB connected to a flexible neural probe with a µLED at the tip (reproduced with permission from [47])

Rossi et al. presented in 2015 one of the first wireless stimulators connected to an implantable probe containing a µLED [30] (Fig. 3a). The probe consisted of a blue (465 nm) bare chip µLED soldered to a reduced-thickness PCB with a SMD connector. The stimulator was composed of a microcontroller with built-in flash and random-access memories, a RF transceiver (2.4–2.5 GHz), and a rechargeable LiPo battery. The communication range between the transmitter and the headstage was 4 m. The maximum µLED optical power achieved was approximately 32 mW, with a reported temperature increase of 0.5 ºC for duty cycles below 20%. The complete device size was 14 × 17 × 5 mm and weighed 2.9 g. Battery lifetime was reported to be 2 h. A software, OptoStim, was also developed to facilitate user definition of stimulation parameters. The device was tested in vivo for striatal stimulation of ChR2 mice with concomitant assessment of motor activity. Although the implantable probe was easily assembled in any dry lab, its width (0.7 mm width) was higher than the larger 500 µm diameter optical fibers used for mice experiments, which could increase tissue damage. This device was later sold commercially by Triangle Biosystems International (Durham, USA) [34], which is no longer operational.

In 2019, Zhao et al. developed a wireless stimulator device with a similar strategy for RF communication and battery power to the one described by Rossi et al. [30], but integrated a thin flexible implantable probe instead [35]. The headstage had an RF antenna to receive the command signals, with a range of 55 m, a microcontroller to decode the signal into stimulation parameters, and a microcontroller chip to convert command signals to constant currents for consistent LED output power. When compared with [30] there was a reduction in the device’s footprint to 10 × 18 mm and in the weight to 1.9 g. Battery lifespan was also higher, with 4 hours of independent operation for 10 mA input current and 20% duty cycle stimulation. The neural probe consisted of a InGaN (indium gallium nitride)-based blue (471 nm) μLED transferred onto a flexible polyimide substrate and coated with an insulation layer consisting of a mix of polyisobutylene and PDMS (polydimethylsiloxane), with a final implantable footprint that was 300 μm wide and 140 μm thick. For an operating current of 5 mA, the μLED delivered up to 82 mW/mm^2^ optical power, while keeping the temperature increase below 1 ºC as long as the frequency and duty cycle were not higher than 20 Hz and 80% respectively. The device was tested in vivo with optical stimulation of the hippocampus and the cuneiform nucleus of Thy1-ChR2 expressing mice during open field exploration. Li et al. presented an updated version of this device in 2022 that included two different co-localized μLEDs with two different colors (blue and red) in the implantable probe [36] (Fig. 3b). The final footprint of the implantable probe was approximately 320 μm wide and 120 μm thick. The blue and red μLEDs had reported irradiances of up to 200 mW/mm^2^ and 50 mW/mm^2^, respectively. In vivo validation of the device was performed in mice with optogenetic stimulation of stGtACR2 and ChrimsonR expressing neurons in the VTA.

Montgomery et al. also presented an early version of a wireless optical stimulator system with an implantable µLED in 2015 [31], that additionally included WPT capabilities by RF resonance (further described by Ho et al. [37]). The headstage device was composed by a simple copper coil and a rectifier circuit permanently connected to an implantable µLED by two twisted 36 AWG magnet wires covered with a thin layer of Parylene C to improve biocompatibility and impermeability. Because acrylic had to be applied to isolate the connection between the wires and the µLED pads, the total thickness of the µLED extension tip of the device was approximately 350 µm. WPT and device control were achieved with an aluminum resonant cavity with a surface lattice of hexagons to couple electromagnetic energy (1.5 GHz) to the coil in the mouse’s headstage (Fig. 3c). Owing to the confined electromagnetic field pattern of the lattice and the size of the mouse, energy was concentrated on the mouse at all positions (self-tracking). This system could provide a time-averaged input power of 3.2 W, at 20% duty cycle, which was below specific absorption rate (SAR) levels and could drive the µLED at the desired output power. Light intensity was measured at distance of 3 cm from the surface lattice and, due to thermal conditions, the maximum irradiance of the implantable µLED was limited to 20 mW/mm^2^ (20% duty cycle) to reduce heating dissipation inside the brain. Nevertheless, the authors reported higher heating for the µLED when compared with optical fibers using the same stimulation parameters. Compared with all other wireless optical stimulators published to that date, this was considerably lighter with a total weight of only 20–50 mg and a total volume of 10 mm^3^. This was possible due to the strong localization of electromagnetic energy at low GHz, as well as to the proximity of the resonant cavity to the headstage. The small weight and size allowed that the headstage was permanently fixed to the skull below the skin. However, the µLED extension tip presented a higher diameter than conventional 100–200 µm diameter optical fibers and behavioral tests could only be performed on top of the resonant cavity, limited to a diameter of 21 cm. The system was tested in vivo in Thy1-ChR2-YFP mice targeting the premotor cortex. Due to its reduced dimensions, its potential for spinal cord and peripheral nerve endings stimulation was also demonstrated in vivo.

Another early wireless optical stimulator using a µLED probe powered and controlled via RF communication was presented by Park et al. in 2015 [38]. The µLED implantable probe fabrication was based on a previous paper showcasing one of the earliest demonstrations of µLEDs integration in a flexible polymeric implantable neural probe [39]. The control module was also fabricated on a flexible polymeric substrate, with all the components mounted on polyimide with photolithographed copper traces. This module was attached to the µLED probe in a polyethylene terephthalate (PET) substrate through a connector and measured 3.5 × 2.4 × 8.5 mm, weighing 0.7 g. A variation of this device including photovoltaic power harvesting through two solar cells was also presented, with approximate dimensions of 5 × 12 mm. With this design variation, the authors show operation powered by a desk lamp placed 20 cm away from the animal, providing sufficient current for a µLED maximum irradiance of 2.25 mw/mm^2^. The same authors also presented in 2016 another wireless headstage with an implantable µLED neural probe, both in flexible silicon elastomer substrate [40]. This device was the first demonstration of a fully integrated system where wireless power and control units were monolithically fabricated with the implantable part containing the µLED. The harvester module worked through RF communication, and the neural probes included 2 µLEDs of different colors controlled by different resonant frequencies (2.3 and 2.7 GHz). The device was designed to be completely implanted below the skin with a 4.3 × 8 × 0.7 mm stretchable PDMS encapsulated circuit, weighing only 33 mg, conforming to the skull. The implantable neural probe was approximately 250 µm wide with a 75 µm thick biodegradable PLGA (poly-lactic-*co*glycolic acid) substrate which dissolved in 3 days following implantation. To improve the efficiency of the WPT, the full system included online video tracking with a camera and automatic selection of the optimal antenna array to direct the maximum power selectively to the animal’s position. The system was tested in vivo by targeting the locus coeruleus deep brain region in Gal-Cre mice.

A device based on a simple miniaturized coil and inductive coupling near-field communication (NFC), with a similar concept to [31, 40], was developed by Shin et al. in 2017 [32] (similar design to device on Fig. 3d). Remote device control and powering relied on the use of high-frequency coupling at 13.56 MHz (instead of GHz ultra-high frequencies, as in [31] and [40]). A RF generator was used to power the device, with a maximum range of 30 cm at 12 W of output power that defined the frequency and duty cycle of the stimulation. The headstage consisted of a small copper coil antenna in a polyimide substrate, a SMD capacitor, a rectifier, and an indicator LED. The implantable portion of the device consisted of a 350 µm wide and 130 µm thick flexible shank with a mounted blue µLED at the tip connected to the coil by a serpentine cable. All parts of the device were encapsulated in PDMS and parylene, with a final thickness up to 500 µm. The maximum optical power reported was 50 mw/mm^2^ for the blue µLED (470 nm), with negligeable temperature increases for irradiances of 20 mW/mm^2^ at any duty cycle. The small size and weight of the headstage part of the device, with a diameter below 10 mm and weight of 30 mg, allowed subdermal implantation in mice. The authors also showed that the µLED light could be changed to other colors relevant to optogenetic stimulation such as green (530 nm), yellow (560 nm) and red (650 nm) with the application of phosphor dyes to the blue µLED. The device was tested in vivo with optical stimulation of the VTA and nucleus accumbens (NAc) in mice during a place preference test. This system is currently sold by Neuralux [41]. In 2018, Gutruf et al. proposed additional design variations to this system that included two fixed implantable shanks each with two different µLEDs to target four different brain areas, and a microcontroller to allow customization of stimulation frequency and duty cycle [42]. Different stimulation protocols were stored in the microcontroller memory and the receiver antenna triggered the pre-defined stimulation. To overcome angle dependency in power transfer to the headstage, reported in [32], the authors also proposed the use of two transmitting antennas oriented orthogonally to each other to improve WPT. In 2021, Ausra et al. [43] presented further variations of the designs described in [32, 40, 42], by replacing the implantable probe with a µLED placed on the skull for transcranial optogenetics and using a capacitor bank in the headstage for energy storage. This alternative weighted 76 mg and had a footprint of 11.73 × 7.95 × 0.39 mm. The µLED used for transcranial stimulation was red colored (628 nm) and presented an irradiance of 226.56 mW/mm^2^. In vivo demonstration of the device was performed with transcranial stimulation of the secondary motor cortex (M2) in ChrimsonR-expressing mice in an open field. In the same year, Yang et al. adapted the previous device to support two independent stretchable neural probes with one µLED each for bi-hemispheric optical stimulation [44]. The µLEDs in the probes were blue (460 nm), green (535 nm), orange (595 nm) and red (630 nm) and each presented different energy-conversion efficiencies (36.1%, 6.6%, 6.4% and 26.7%, respectively). This version presented two options: a head-mounted and a back-mounted device, with footprints of 10 × 12 mm and 11 × 19 mm, respectively. The stimulation parameters were independently controlled with a GUI via NFC. In vivo validation was performed in a social behavior paradigm with stimulation of the medial prefrontal cortex (mPFC) in ChR2-expressing mice.

In recent years, projects using Bluetooth Low Energy (BLE) modules for wireless communication in biomedical devices have gained popularity [45], and have also been integrated in wireless optical stimulations. This preference is due to various advantages of BLE including the long operational distance, low power consumption, and bidirectional communication that enables the design of closed-loop systems. In 2020, Orguc et al. proposed a device with an optic fiber based implantable probe that carried two μLEDs for optical stimulation and was controlled wirelessly by a BLE chip [46]. The headstage consisted of a 14 mm diameter circular PCB with a BLE system-on-a-chip (SoC), a coin rechargeable lithium battery, and a J-Link pin interface for chip programming. The implantable probe was connected to the headstage by pin connectors, and μLED irradiance was 4.97 mW/mm^2^. The device weighed 2.2 g and battery lifespan was estimated to be approximately 12 h for 1 s stimulation periods every 4 s at 20 Hz and 20% duty cycle. To control the light output irradiance, the users could add an additional control module that increased the device’s weight to 3.2 g. Communication with the headstage was not affected by the mouse’s head orientation, in contrast with RF solutions. The headstage was tested in vivo with stimulation of the VTA in Thy1-ChR2 expressing mice.

A more recent device using BLE for wireless control and powered by a battery recharged by solar power was presented by Park et al. in 2023 [47] (Fig. 3e). The solar cell was connected to a power management circuit that provided current to a rechargeable LiPo battery (< 0.4 g) and a BLE SoC module for communication. Commercial mobile user interfaces could be used to communicate with the BLE chip that controlled the μLED stimulation parameters. The device was fabricated on a polyimide flexible substrate with all components, including the fabricated solar cell, sitting on top of copper traces. Device passivation was achieved with a silicon elastomer. Final headstage size was approximately 35 x 10 mm, but it could fold to be approximately 15 × 10 mm with the solar cell sitting on top. The implantable part of the device consisted of an implantable shank permanently attached to the headstage on flexible polyimide and a μLED at the tip that could irradiate blue light (460 nm) up to 40 mW/mm^2^. Implant footprint was approximately 150 μm wide and at least 140 μm thick. The measured temperature changes induced by optical stimulation were below 1 ºC only for frequencies of 20 Hz and a 20% duty cycle. The battery could last for up to 4 days with the μLED turned off. In vivo validation was performed by M2 opto-stimulation of Thy1-ChR2-YFP mice during locomotor behavior.

### Multifunctional Optical Stimulators

The progressive miniaturization of electronic components and fabrication techniques with micro-/nano-scale resolution have enabled the integration of novel functionalities in neural interfaces. Thus, in addition to delivering light to the brain some wirelessly-controlled headstages have also incorporated tools for electrophysiological recordings, temperature and neurotransmitter sensing, and pharmacological delivery. This multifunctionality significantly maximizes the potential of implanted devices, enabling a more comprehensive study of brain circuits and the development of novel closed-loop systems.

The first wireless multifunctional device integrating optical stimulation was presented by Kim et al. in 2013 and combined light delivery through µLEDs with temperature sensing and one electrode for electrophysiological recordings [39]. The control and powering of the device resorted to RF, which modulated the electrical input power and, consequently, the μLED (450 nm) optical power output. The headstage, integrating a control module, was fabricated in a flexible polyimide or a rigid PCB, weighing approximately 0.7 g and 2 g, respectively. The implantable probe containing an array of four gallium nitride µLEDs, a platinum (Pt) electrode, and a serpentine Pt resistor for temperature sensing, and measuring 390 μm-width and 20 μm-thickness, connected to the headstage through a pin connector. At 1 m from the RF transmitter, the μLEDs presented an irradiance of 7 mW/mm^2^, and temperatures below 1ºC for frequencies up to 10 Hz (10% duty cycle). This device was tested in vivo, in ChR2-YFP mice implanted in the VTA during a reward behavioral paradigm.

**Fig. 4:**
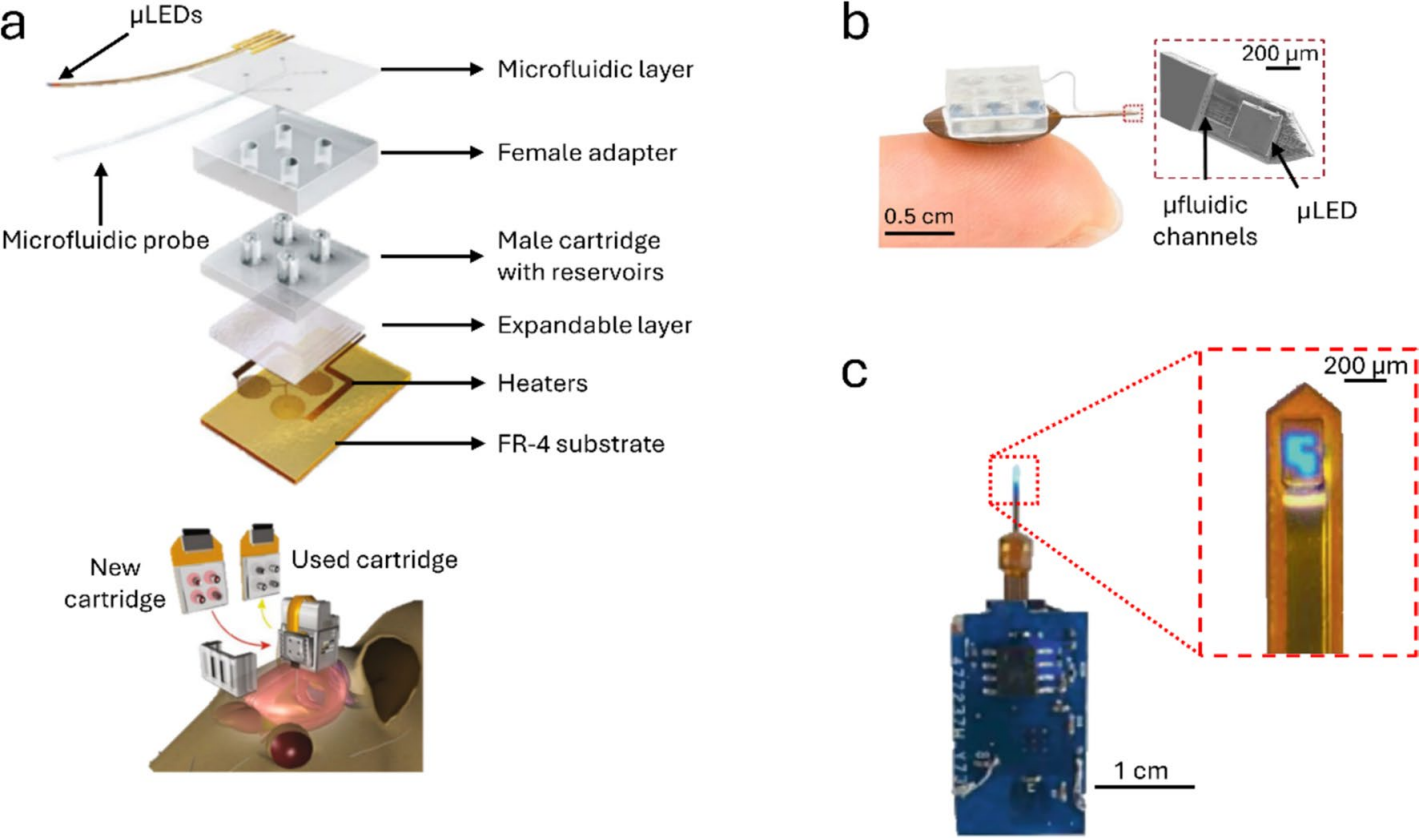
Multifunctional wireless optical stimulators. **a** Wireless device that combines optical stimulation with pharmacological delivery, controlled and powered by NFC. The headtage contains replaceable drug cartridges that can be changed between experiments. The implantable neural probe includes a µLED for optical stimulation and PDMS microfluidic channels for drug delivery (reproduced with permission from [50]). **b** A variation of the probe in **a** with refillable reservoirs (from [51] (PNAS License)). **c** Multifunctional device for optical stimulation and electrochemical dopamine sensing. The headstage is a PCB with a RF transceiver, a LED driver, a digital-to-analog converter (DAC) and pre-amplifiers for voltammetry measurements. The flexible implantable neural probe contains a blue µLED for optical stimulation and a PEDOT:PSS-coated diamond working electrode for voltammetry (the reference and counter electrodes of the voltammetry cell were external platinum and Ag/AgCl wires) (adapted from [52] (CC-BY))

Jeong et al. presented in 2015 the first wireless headstage for mice that combined optogenetics with fluidics for pharmacology applications [48]. This battery-powered IR wireless device was able to not only stimulate deep brain regions with monochromatic light but also deliver different pharmacological agents to a specific brain area, surpassing limitations intrinsic to the tethered combination of optogenetic and pharmacologic techniques with optical fibers and rigid tubing. A flexible implantable PDMS neural probe was fabricated with four microfluidic channels and four μLEDs measuring 500 × 56.5 μm (width × thickness). The headstage integrated an IR receiver with four independent liquid reservoirs, sealed with copper membranes, each connected to an independent microfluidic channel on the implantable portion of the device. Fluidic delivery was achieved with the inclusion of an expandable layer and a Joule heater layer, fabricated with serpentine traces of gold, where the heating of the later would lead to the irreversible expansion of the first layer pushing the liquid into the channels. Although the reservoir heated up to 105 ºC during this process, the fluid cooled down as it travelled through the microchannels, targeting the tissue with a temperature not over 0.1 ºC that of the tissue. Two 3.7 V batteries (0.33 g each) were connected to the headstage for power. The device with a final volume of 1575 mm^3^ and a total weight of approximately 1.85 g was implanted in mice and tested in a place preference experiment with stimulation of the NAc. Improvements to this device were presented in later years. In 2018, Noh et al. created a battery-free version which included an RF energy-scavenging circuit with two antennas and only one reservoir for liquids [49]. The exclusion of the batteries and managing circuits presented significant reductions in weight (220 mg) and volume (125 mm^3^) compared to [48]. At 10 cm from the RF transmitter the optical irradiance at the tip of the implantable probe was close to 30 mW/mm^2^. On [48, 49] the devices’ reservoirs could only be used once, thus Qazi et al. presented another version of the device in 2019 now capable of chronic drug delivery [50]. The drug infusion strategy was similar, but instead of fabricating the microfluidic channels permanently connected to the reservoirs this version permitted attachment of the reservoirs to the headstage only during experiments (Fig. 4a). The implantable neural probe had four microfluidic channels and two μLEDs—blue (470 nm) and orange (589 nm). The device, weighing approximately 2 g, integrated a battery for power and a BLE chip for communication up to 100 m with a smartphone app that controlled stimulation parameters. This device was implanted on animals targeting the VTA. Another version with refillable reservoirs was proposed by Zhang et al. in 2019 [51] which, unlike [50], was wirelessly powered and controlled by NFC 13.56 MHz (Fig. 4b). The thermal operation reservoirs from [48] were also replaced with an electrochemical micropump, preventing the irreversible deformation of the reservoirs. Re-filling between experiments was performed with a syringe, and the reservoirs were re-sealed with silicone or paraffin wax. Compared with [48], this headstage was lighter (0.29 g) but it was larger, with a radius and thickness of 0.5 cm and 0.4 cm, respectively. In vivo validation was done by targeting the VTA in mice for a motor behavior paradigm.

Liu et al. proposed a multifunctional wireless device that included optogenetic stimulation and electrochemical dopamine sensing in 2020 [52] (Fig. 4c). The device consisted of a PCB with a transceiver operated at 2.4 GHz, a LED driver chip, a digital-to-analog converter and pre-amplifiers for voltammetry measurements. The headstage had a footprint of 2.2 × 1.3 cm and a weight of 2.0 g and was powered by a rechargeable lithium ion-battery contributing with 0.9 g to the total weight. The implantable portion of the system consisted of a flexible double-sided copper polyimide shank with an InGaN µLED (470 nm) and a Poly(3,4-ethylenedioxythiophene) polystyrene sulfonate (PEDOT:PSS)-coated diamond electrode that served as the electrochemical sensor. The voltammetry cell, besides the PEDOT-PSS working electrode, consisted of reference and counter electrodes based on silver/silver chloride (Ag/AgCl) and Pt wires, respectively, which were implanted separately. The total dimensions of the implantable probe were 360 µm × 90 µm (width x thickness). Although irradiance data was not provided for the µLED, temperature increases over 1 ºC were reported for low driving currents (> 2 mA) at 20 Hz and 20% duty cycle. In vitro testing of dopamine electrochemical sensing with chronoamperomety operation showed a limit of detection of approximately 0.5 µM with a detection sensitivity of 0.06 nA/μM, which is in line with other voltametric dopamine sensors [53]. This device was tested in a real-time place preference paradigm, targeting the VTA in adult mice.

### Concluding Remarks

In this review, we critically described different devices for optogenetics in freely moving mice that can be wirelessly controlled. Over the past decade, researchers took advantage of electronic components’ miniaturization and efficiency improvements to continuously reduce device sizes and increase functionalities. In terms of wireless communication strategies most devices used IR and RF technologies, such as Wi-Fi, NFC and Bluetooth. While each may have advantages and disadvantages concerning working range, price, bulkiness and weight, devices using recent SoC approaches could more easily integrate advanced microcontroller functions and low-power communication strategies such as BLE.

When it comes to powering the device, the strategies used were either battery supply or WPT through near-field magnetic resonant coupling. The continuous introduction of smaller and longer-lasting batteries contributed to improving operating time in devices resorting to independent power. However, batteries are heavy and bulky and contributed to a significant part of the total weight and footprint of the reviewed devices. On the other hand, optical stimulators powered through WPT, although lighter and frequently fully implantable subdermally, were limited to working ranges below one meter and relied on complicated approaches to improve the low coupling efficiency. Advances in mid-field and far-field WPT may create novel opportunities to extend operating ranges, and the combination of WPT and battery storage, including the use of more efficient and smaller supercapacitors, may offer opportunities for longer lasting operation times.

The introduction of micron-size LEDs has also played an important role in the miniaturization and power efficiency of wireless stimulators by allowing the replacement of bulky and power-hungry laser sources. These advances were accompanied by improvements in the realization of micron-sized silicon and polymer-based optoelectronic implantable neural probes with µLEDs that are suited for long-term biointegration. As the number of devices using µLEDs grew, there was also additional room to integrate other technologies for true multifunctional devices. The number of wireless devices combining optogenetics with other functionalities such as electrophysiology recordings or neurotransmitter sensing is still small, but it is set to grow more significantly over the next years especially as they are used in closed-loop experiments.

## Data Availability

Data sharing is not applicable to this article as no new data were created or analyzed in this study.

## Abbreviations

AC: Alternating current
Ag/AgCl: Silver/silver chloride
BLE: Bluetooth low energy
ChR2: Channelrhodopsin-2
DAC: Digital-to-analog converter
DC: Direct current
DIP: Dual in-line package
GUI: Graphical user interface
HDPE: High-density polyethylene
InGaN: Indium gallium nitride
IR: Infrared radiation
LED: Light-emitting diode
LiPo: Lithium polymer-based
M2: Secondary motor cortex
mPFC: Medial prefrontal cortex
NAc: Nucleus accumbens
NFC: Near-field communication
PEDOT:PSS: Poly(3,4-ethylenedioxythiophene) polystyrene sulfonate
PCB: Printed circuit board
PDMS: Polydimethylsiloxane
PWM: Pulse width modulation
Pt: Platinum
RF: Radiofrequency
SAR: Specific absorption rate
SMD: Surface-mount device
SoC: System-on-a-chip
SWD: Serial wire debug
Thy: Thymus cell antigen
VTA: Ventral tegmental area
WPT: Wireless power transfer
YFP: Yellow fluorescent protein
µLED: Micro-scale light-emitting diode
